# II Brazilian Consensus on the use of human immunoglobulin in patients with primary immunodeficiencies

**DOI:** 10.1590/S1679-45082017AE3844

**Published:** 2017

**Authors:** Ekaterini Simões Goudouris, Almerinda Maria do Rego Silva, Aluce Loureiro Ouricuri, Anete Sevciovic Grumach, Antonio Condino, Beatriz Tavares Costa-Carvalho, Carolina Cardoso de Mello Prando, Cristina Maria Kokron, Dewton de Moraes Vasconcelos, Fabíola Scancetti Tavares, Gesmar Rodrigues Silva Segundo, Irma Cecília Douglas Paes Barreto, Mayra de Barros Dorna, Myrthes Anna Maragna Toledo Barros, Wilma Carvalho Neves Forte

**Affiliations:** 1 Universidade Federal do Rio de Janeiro, Rio de Janeiro, RJ, Brazil.; 2 Universidade Federal de Pernambuco, Recife, PE, Brazil.; 3 Hospital Servidores do Estado do Rio de Janeiro, Rio de Janeiro, RJ, Brazil.; 4 Faculdade de Medicina do ABC, Santo André, SP, Brazil.; 5 Universidade de São Paulo, São Paulo SP, Brazil.; 6 Universidade Federal de São Paulo, São Paulo, SP, Brazil.; 7 Hospital Infantil Pequeno Príncipe, Curitiba, PR, Brazil.; 8 Hospital das Clínicas, Faculdade de Medicina, Universidade de São Paulo, São Paulo, SP, Brazil.; 9 Hospital de Base do Distrito Federal, Brasília, DF, Brazil.; 10 Universidade Federal de Uberlândia, Uberlândia, MG, Brazil.; 11 Centro Universitário do Estado do Pará, Belém, PA, Brazil.; 12 Faculdade de Ciências Médicas, Santa Casa de São Paulo, São Paulo, SP, Brazil.

**Keywords:** Immune system diseases, Immunoglobulins, Immunoglobulins, intravenous, Immunologic deficiency syndromes, Immunization, passive

## Abstract

In the last few years, new primary immunodeficiencies and genetic defects have been described. Recently, immunoglobulin products with improved compositions and for subcutaneous use have become available in Brazil. In order to guide physicians on the use of human immunoglobulin to treat primary immunodeficiencies, based on a narrative literature review and their professional experience, the members of the Primary Immunodeficiency Group of the Brazilian Society of Allergy and Immunology prepared an updated document of the 1^st^ Brazilian Consensus, published in 2010. The document presents new knowledge about the indications and efficacy of immunoglobulin therapy in primary immunodeficiencies, relevant production-related aspects, mode of use (routes of administration, pharmacokinetics, doses and intervals), adverse events (major, prevention, treatment and reporting), patient monitoring, presentations available and how to have access to this therapeutic resource in Brazil.

## RESUMO

Nos últimos anos, novas imunodeficiências primárias e defeitos genéticos têm sido descritos. Recentemente, produtos de imunoglobulina, com aprimoramento em sua composição e para uso por via subcutânea, tornaram-se disponíveis em nosso meio. Com o objetivo de orientar o médico no uso da imunoglobulina humana para o tratamento das imunodeficiências primárias, os membros do Grupo de Assessoria em Imunodeficiências da Associação Brasileira de Alergia e Imunologia produziram um documento que teve por base uma revisão narrativa da literatura e sua experiência profissional, atualizando o I Consenso Brasileiro publicado em 2010. Apresentam-se novos conhecimentos sobre indicações e eficácia do tratamento com imunoglobulina nas imunodeficiências primárias, aspectos relevantes sobre a produção, forma de utilização (vias de administração, farmacocinética, doses e intervalos), efeitos adversos (principais efeitos, prevenção, tratamento e notificação), monitorização do paciente, apresentações disponíveis e forma de obtenção deste recurso terapêutico em nosso meio.

## ABOUT THIS PAPER

This document was collaboratively prepared by members of the Primary Immunodeficiency Group of the Brazilian Society of Allergy and Immunology, based on a narrative literature review and their professional experience, to guide Brazilian physicians on the use of human immunoglobulin (Ig) to treat primary immunodeficiencies.^1^

## INTRODUCTION

Primary immunodeficiencies (PID) are a very heterogeneous group currently made up of more than 300 diseases caused by genetic mutations, leading to abnormal development and function of the immune system, and are characterized by recurrent infections (either severe or caused by unusual or low-pathogenicity agents), autoimmune or inflammatory manifestations, and a greater predisposition to cancer.^2-6^ In the past few years, new diseases and new genetic defects have been described.

Several registries around the world, including the Latin American, show that at least 50% of PIDs predominantly affect antibody production, which is also impaired in other types of immune system defects.^3,7-13^

The use of serum from animals or convalescent humans in the treatment and prevention of infectious diseases started at the end of the 19^th^ century. Purification of Immunoglibulin G (IgG) became possible in the 1940’s with the Cohn-Oncley fractionation method,^14^ used to produce albumin for the injured during World War II.^15,16^

Human Ig replacement therapy was performed for the first time in 1952, by Bruton, in the first patient described with agammaglobulinemia, using the subcutaneous route.^17^ In the years that followed, the intramuscular route became the most widely used for Ig replacement. However, this mode of administration is painful, reaches serum concentrations in about 24 hours, and has low bioavailability and less than 50% recovery.^18^ With the use of higher doses, side effects, such as chills, fever or even anaphylaxis, occur more frequently.^15^

Since the 1960’s, different preparations for intravenous administration have been developed and put to use, but it was only in the late 1970’s and early 1980’s that this route became the route of choice for Ig replacement in PID patients.^16,18,19^

In the 1980’s, improvements were made to the production process and composition of this immunobiological for intravenous infusion, allowing the use of higher Ig doses with better infection control, but still with many adverse effects.^18,20^ At that point in time, subcutaneous administration started to be reported by several services.^21-27^ It has been increasingly used in the last 10 to 15 years, with good clinical results, few adverse effects and other advantages when compared to intravenous, as described later.^28-42^ Products for subcutaneous use have been available in Brazil since 2015.

In the context of major advancements in the knowledge of PIDs and the production of human Ig, and with new products available in the market, we must update the first consensus published in 2010, which is currently in use in Brazil.

## OBJECTIVE

To update the 1^st^ Brazilian Consensus on the Use of Human Immunoglobulin in Patients with Primary Immunodeficiencies, published in 2010. The text presents advancements in knowledge of indications and efficacy of Ig replacement in primary immunodeficiencies, in addition to relevant facts about production, mode of use (administration routes, pharmacokinetics, doses and intervals), adverse events (major effects, prevention, treatment and reporting), patient monitoring, presentations available and how to have access to this therapy in Brazil. The use of human Ig in secondary immunodeficiencies or as an immunomodulator in autoimmune and inflammatory diseases is not addressed in this paper.

## METHODS

A foundation text was prepared by the coordinators of the advisory group, based on scientific publications on the use of Ig in primary immunodeficiencies in the last 10 years, retrieved from PubMed and Google Scholar, as well as relevant textbooks and guidelines, in the form of a narrative literature review.

The text was sent by e-mail to the other 14 members of the group, to be expanded and modified so as to reflect the technical and literature-based knowledge as well as the clinical experience of all involved.

A final review of the text was carried out by two specialists in the field who were not part of the group.

## INDICATIONS AND EFFICACY OF HUMAN IMMUNOGLOBULIN IN PRIMARY IMMUNODEFICIENCIES

Treatment with Ig is currently the leading therapeutic approach in almost 75% of PDIs, *i.e*. those in which antibody production is impaired,^5,43^ promoting the replacement of immunoglobulin G or IgG. The objectives are to maintain stable and adequate serum concentrations of this type of Ig and achieve good clinical management of patients.^44-48^

The target serum IgG concentration had been set at 500mg/dL in blood samples collected immediately before the infusion,^44,49-52^ but the clinical monitoring of patients has shown that higher values, approximately 700 to 1,000mg/dL, are more efficient to control infections, particularly pneumonia.^8,49,53-60^ Higher target IgG concentrations are especially important in patients with chronic pulmonary disease and bronchiectasis, promoting improved lung function.^55,61-64^

It is important to note that the IgG concentrations required for infection prevention vary among individuals, and the treatment must be individualized to find the doses and serum IgG concentrations leading to good clinical responses in each patient (also known as biological IgG level).^8,38,45,55,56,58,60,65-67^ “Good clinical control” is defined as a decrease in the number and severity of infectious and inflammatory conditions, and a decrease in hospitalizations and use of antibiotics, preventing certain complications and improving general health and quality of life.^59,67-71^ In patients with normal IgG concentrations before initiating treatment (specific antibody deficiencies, for example), the clinical response alone is used for adjustment of Ig therapy.^67^

The recommendations of the European Society for Primary Immunodeficiencies (ESID) regarding human Ig replacement are^72,73^ serum IgG<200mg/dL is always an indication, except for patients with transient hypogammaglobulinemia of infancy with no severe infections; serum IgG between 200 and 500mg/dL is an indication in case of antibody production deficiency or recurrent and/or severe infections; serum IgG>500mg/dL is an indication for Ig replacement only when abnormal production of specific antibodies is verified, and recurrent and severe infections are present.

According to these recommendations, the use of human Ig is indicated in all PIDs in case of documented impairment in the production of IgG antibodies.^47,74^

However, there are evidence-based indications in some PIDs: abnormal antibody production related to B-cell defects (X-linked agammaglobulinemia, common variable immunodeficiency, defective production of specific antibodies, defects of IgG subclasses with abnormal antibody production), except for selective IgA deficiency, as well as combined immunodeficiencies with or without associated syndromes (severe combined immunodeficiencies, X-linked hyper-IgM syndrome, X-linked lymphoproliferative syndrome, Wiskott- Aldrich syndrome, NEMO deficiency, Warts syndrome, WHIM syndrome - warts, hypogammaglobulinemia and immunodeficiency), and after hematopoietic stem cell transplant in PID patients.^47,74-79^ There is some evidence of benefits in hyper-IgE syndrome, ataxia-telangiectasia, DiGeorge syndrome and anticytokine-autoantibody-mediated disorders (Chart 1).^78,79^ The immunoglobulin may also be used as an immunomodulator, at higher doses, to treat autoimmune manifestations associated with some PIDs, such as thrombocytopenia or hemolytic anemia.^74,80,81^

**Chart 1:** Primary immunodeficiencies in which immunoglobulin replacement is indicated

Ig indication level	Primary immunodeficiencies
Required and immediate start	X-linked and autosomal recessive agammaglobulinemia
	Common variable immunodeficiency
	Severe combined immunodeficiencies
	X-linked and autosomal recessive hyper-IgM
	Wiskott-Aldrich syndrome
	NEMO deficiency and IKKB
	WHIM syndrome
	Reticular dysgenesis
Depends on confirmation of diagnosis and severity of clinical condition	IgG subclass deficiency
	Specific antibody deficiency
	X-linked lymphoproliferative syndrome
Possible	Transient hypogammaglobulinemia of infancy*
	Ataxia-telangiectasia
	DiGeorge syndrome
	Hyper-IgE syndrome
	IgA + IgG2 and/or IgG4* deficiency

Source: Abolhassani H, Asgardoon MH, Rezaei N, Hammarstrom L, Aghamohammadi A. Different brands of intravenous immunoglobulin for primary immunodeficiencies: how to choose the best option for the patient? Expert Rev Clin Immunol. 2015;11(11):1229-43. Review;^(5)^ Albin S, Cunningham-Rundles C. An update on the use of immunoglobulin for the treatment of immunode ciency disorders. Immunotherapy. 2014;6(10):1113-26. Review;^(78)^ Bonilla FA, Khan DA, Ballas ZK, Chinen J, Frank MM, Hsu JT, Keller M, Kobrynski LJ, Komarow HD, Mazer B, Nelson RP Jr, Orange JS, Routes JM, Shearer WT, Sorensen RU, Verbsky JW, Bernstein DI, Blessing-Moore J, Lang D, Nicklas RA, Oppenheimer J, Portnoy JM, Randolph CR, Schuller D, Spector SL, Tilles S, Wallace D; Joint Task Force on Practice Parameters, representing the American Academy of Allergy, Asthma & Immunology; the American College of Allergy, Asthma & Immunology; and the Joint Council of Allergy, Asthma & Immunology. Practice parameter for the diagnosis and management of primary immunodeficiency. J Allergy Clin Immunol. 2015;136(5):1186-205.e1-78. Review.^(79)^

* In the face of major infections.

WHIM: warts, hypogammaglobulinemia and immunodeficiency syndrome; IgE: immunoglobulin E; IgA: immunoglobulin A; IgG: immunoglobulin G.

Despite having increased in recent years, the use of Ig in patients with secondary hypogammaglobulinemia (Chart 2)^53,82-84^ must be further investigated and is indicated in case of lower levels of serum IgG and/or documented impairment of antigen-specific antibody production and/or presence of relevant infections.^43,74,85^ A condition that has become more frequent in recent years is hypogammaglobulinemia associated with the use of rituximab, an anti-CD20 monoclonal antibody indicated for some autoimmune diseases, lymphoproliferative syndromes, or refractory nephrotic syndrome. This type of hypogammaglobulinemia affects up to 50% of patients, especially those on regular use of rituximab; is symptomatic in less than 10% of cases, and can persist for a long time^86-89^ requiring Ig replacement, either intravenous or subcutaneous.^90^ Persistent hypogammaglobulinemia may occur in a small group of genetically predisposed patients on rituximab.^91,92^

**Chart 2:** Causes of secondary hypogammaglobulinemia

Disease-related	
B-cell disorders	Multiple myeloma, chronic lymphocytic leukemia, Hodgkin's and non-Hodgkin's lymphoma
Protein-losing disorders	Nephrotic syndrome, protein-losing enteropathy and large burns
Lymphatic circulation-related diseases	Intestinal lymphangiectasis, chylothorax, and Proteus syndrome
Infectious diseases	HIV (in children), congenital infections due to rubella, cytomegalovirus, Epstein-Barr virus and toxoplasmosis
Diseases related to increased immunoglobulin catabolism	Myotonic dystrophy and hypersplenism

**Secondary to the use of drugs**	

Immunosuppressants	Corticosteroids, cyclophosphamide, acetyl mycophenolic acid, and cyclosporine
Anticonvulsants	Carbamazepine, phenytoin, lamotrigine and sodium valproate
Immunobiologicals	Rituximab, belimumab, imatinib, dasatinib, and atacicept
Other drugs	Fenclofenac, chloroquine, captopril, sulfasalazine, gold salts, chlorpromazine and D-penicillamine

Source: Rose ME, Lang DM. Evaluating and managing hypogammaglobulinemia. Cleve Clin J Med. 2006;73(2):133-7, 140, 143-4. Review;^(53)^ Grimbacher B, Schäffer AA, Peter HH. The genetics of hypogammaglobulinemia. Curr Allergy Asthma Rep. 2004;4(5):349-58. Review;^(82)^ Garcia-Lloret M, McGhee S, Chatila TA. Immunoglobulin replacement therapy in children. Immunol Allergy Clin North Am. 2008;28(4):833-49, ix. Review;^(83)^ Dhalla F, Misbah SA. Secondary antibody deficiencies. Curr Opin Allergy Clin Immunol. 2015;15(6):505-13. Review.^(84)^

Numerous studies showed a reduction in infections and mortality rates, and an overall improvement of health status and quality of life promoted by intravenous Ig replacement in PID patients.^9,44,46-48,93-97^

We searched the literature and found a number of studies with similar or even better results with the use of subcutaneous Ig, especially in regard to improved quality of life.^20,29,31,34,35,41,42,56,59,68,72,98-104^ This route has also been shown effective and safe in children,^31,37,105-109^ elderly (even those on anticoagulation and antiplatelet therapy),^30,69,110^ pregnant women^69,111^ and obese patients,^112,113^ at the same dose recommended for intravenous use.

Although replacement therapy with human Ig showed to be effective in a specific group of PIDs, it must also be considered for other PIDs in case of documented impairment of antibody production and presence of recurrent and/or severe infections. This therapy is safe and effective when administered either intravenously or subcutaneously.

## PRODUCTION

Ever since the first method for plasma protein fractionation using ethanol was introduced by Cohn-Oncley in the 1940’s,^14^ a series of improvements have been made to the production of Ig, leading to enhanced safety and tolerability.^114^ This process allowed for higher doses to be used intravenously with better clinical management of patients.

The immunoglobulin is purified from human plasma obtained from thousands of donors, ensuring a broad spectrum of protective antibodies. On the other hand, this could increase the theoretical risk for transmission of blood-borne pathogens, but this risk is eliminated by quarantining the donated blood and applying multiple purification steps. Different manufacturers use different combinations of precipitation, filtration, and chromatography to improve product purity (reaching an IgG concentration over 95%).^115,116^ The diverse preparations also contain a small amount of IgA and traces of IgM.

The products available differ in their physicochemical characteristics (presentation, concentration, osmolarity and pH) and excipients (preservatives and IgG aggregation inhibitors).^5,116,117^ The latest products are safe from the standpoint of infection transmission; they are stabilized with amino acids rather than sugars, have lower sodium concentrations and IgA content under 50mg/ml.^5,16,48,114^

The immunoglobulins are not generic products. The characteristics of each product must be considered at the time of prescription, as shown below, and switching must be avoided, except when indicated by the physician.^70,116,118^

## MODE OF USE (ADMINISTRATION ROUTES, DOSES AND INTERVALS)

Human Ig can be administered as intramuscular, intravenous and subcutaneous injections. Considering the rate of adverse effects and the limited volume that can be used, the intramuscular route is no longer used.

The standard loading dose of intravenous Ig is 400 to 600mg/kg/dose, starting at every 21 days.^5,47,119^ The relation between intravenous Ig doses, serum IgG concentrations and clinical control was demonstrated in several studies,^59^ but we must remember that the metabolism of the administered IgG varies among different individuals.^39,49,50,80,120^ Considering the importance of individualizing therapy, the doses and infusion intervals must be adjusted according to the clinical response and IgG concentrations obtained for each patient.^80,116,121^ Higher doses between 600 and 800mg/kg/dose (or up to 1,200mg/kg) may be required and are particularly indicated in case of chronic lung and/or sinus disease.^16,55,56,61,65,94,96,116^ Moreover, Ig is better metabolized during infections and autoimmune/inflammatory diseases, as well as with losses due to comorbidities or PID complications, or yet, in patients with neonatal Fc receptor promoter gene polymorphisms.^38,39,41^ Therefore, greater doses of Ig may be required, even if temporarily, in acute infections (increased IgG catabolism), severe and/or persistent diarrhea (gastrointestinal loss) or hypersplenism (sequestration).^16,80^

**Figure 1:**
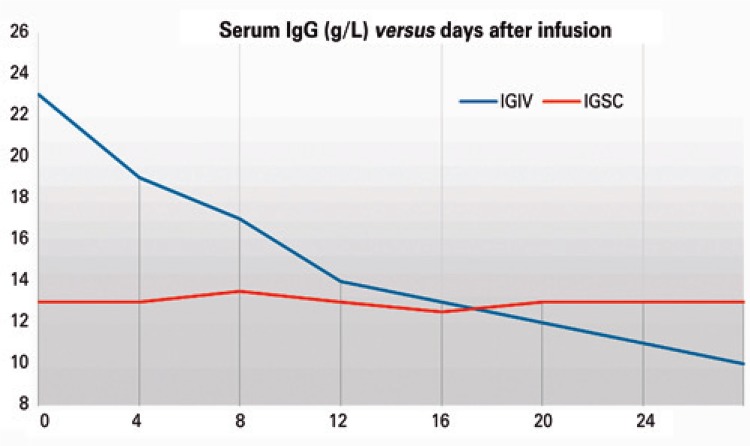
Post-infusion serum concentration of immunoglobulin G in patients on regular use of human immunoglobulin. Comparison of intravenous and subcutaneous immunoglobulin

Source: modified from Wasserman RL, Melamed I, Nelson RP Jr, Knutsen AP, Fasano MB, Stein MR, et al. Pharmacokinetics of subcutaneous IgPro20 in patients with primary immunodeficiency. Clin Pharmacokinet. 201150(6):405-14.^(124)^

IgG: immunoglobulin G; IVIg: intravenous immunoglobulin; SCIg: subcutaneous immunoglobulin.

Increase of serum concentrations with intravenous administration occurs within a few hours at about 100 to 200mg/dL for every 100mg/kg immunoglobulin administered, decreasing rapidly through tissue redistribution within the first few days, with half life around 21 to 28 days (Figure 1).^51,56,120,122^ As good clinical control and stable serum IgG concentrations are established, intravenous Ig infusions can be performed every 28 days.^42,97,123^ Stable IgG values are usually achieved within 3 (or up to 6) months of infusions.^47,51^

Patients with very low IgG concentrations (<200mg/dL) are initially treated with intravenous Ig, often at initial doses of 800 to 1,000mg/kg, leading to a faster increase in serum concentrations.^116^

Subcutaneous Ig is used at the same dose as intravenous Ig − about 400 to 600mg/kg/month, *i.e*., approximately 100 to 150mg/kg every week.^42,56,59^ The increase in serum IgG concentrations was estimated at 84.4mg/dL for each 100mg/kg/month increase in the subcutaneous Ig dose.^59^ Blood IgG concentrations increase less rapidly than with intravenous infusions,^123^ peaking at 2 to 4 days.^51,125^ When starting subcutaneous administration, shorter intervals are recommended as follows: 100mg/kg for 5 consecutive days in the first week,^126,127^ or twice-weekly in the first 2 weeks.^128,129^ Serum IgG concentrations are more stable with subcutaneous Ig^130^ and reached within 6 to 10 weeks of use (Figure 1).^124,125^ The interdose interval may go from fortnightly to daily, using infusion pumps or push*.* ^67,109^ There has been recent evidence showing that higher doses of subcutaneous Ig, as has been established for intravenous Ig, are related with superior clinical control of patients.^42,59^

In patients who wish to switch from intravenous to subcutaneous, we must use a quarter of the previous monthly dose, starting subcutaneous infusions one to two weeks after the last intravenous infusion.^29,59,67,100,104,127,131^

Subcutaneous administration should be preferably in the abdomen, but can also be given in the arms or thighs,^132^ without the need for site rotation.^32^ Local skin hygiene must be performed with alcohol or chlorhexidine. Local anesthetics or ice may be applied to reduce pain if necessary.^132^ There is no need to use gloves, but proper hand washing is critical.^132^ The infusion can be administered in two to four sites, simultaneously or sequentially, weekly or every two weeks.^132,133^ When using infusion pumps, the infusion rate must be 0.1 to 0.25mL/kg/hour/site, reaching up to 15mL/hour/site initially, and then a maximum of 25mL/hour/site.^67^ In case of more frequent administration by push, doses can be daily, twice or three times per week, or even weekly, with adequate safety and a shorter administration time when compared with pumps.^67,134,135^ Administration requires 1 to 10mL syringes with 25 to 23 gauge wing sets, 4 to 6mm needles for children and 9 to 15mm needles for adults,^108,132,136^ or special perpendicular needles,^26,137^ at a rate of 1ml/minute.^35^ The total volume applied per site depends largely on the individual tolerability and also varies according to the product, dose and administration time.^67,135,136^ In children, depending on the weight and age group, it is usually possible to give up to 10 to 20mL per site, whereas in adults, 30 to 40mL/site or up to 80mL, in some cases.^27,48,67,132,134,135^ A recent survey in Europe showed that most patients receive about 20mL per site with good tolerability.^134,138^

A new product for subcutaneous use, already commercially available though not in Brazil, includes the application of hyaluronidase first and then (10 minutes later) the Ig solution, using the same route, allowing for a higher infusion volume per site.^139,140^ In this setting, it is possible to inject subcutaneous Ig every 21 to 28 days, just like intramuscular injections, with appropriate safety and good clinical results.^19^ Doses must be gradually increased over 7 weeks, which limits the use of this product when initial IgG levels are too low (<200mg/dL). In countries where this product is available, it has not yet been released for use in pregnant women and patients under 18 years of age.^67^

Studies indicate that Ig therapy is safe during pregnancy, both intravenously or subcutaneously.^111,141^ Doses should be increased according to the clinical control and serum IgG concentrations achieved. Although the IgG given to pregnant women can cross the placenta and passively protect the fetus, the dose must be increased (20 to 30%) in the last trimester of pregnancy, to ensure adequate levels of antibodies to the newborn.^111,119,142^

Therapy must continue throughout the patient’s life, except in those subjected to hematopoietic stem cell transplantation, and in patients with unspecified hypogammaglobulinemia, who can regain the ability to produce Ig.^74,79,119^ In those cases, infusions can be given at increasingly longer intervals, with close monitoring of the patient and serum IgG values, until they can be discontinued.^143^ However, there is no consensus in the literature on how to proceed in these situations.

## ADVERSE EFFECTS, PREVENTION, TREATMENT AND REPORTING

Treatment with Ig is quite safe, but adverse effects have been described in 1 to 81% of patients or infusions; 30 to 40% of patients; and 5 to 15% of infusions.^144^ They can be mild, moderate or severe,^145,146^ immediate (during or shortly after infusion) or late (hours to days after infusion).^147^ Those considered mild do not cause any changes to vital signs and are resolved with symptomatic drugs without the need to stop the infusion. If signs and symptoms progress and/or persist requiring interruption of the infusion, the adverse effects are considered moderate. Severe adverse effects require immediate discontinuation of the drug and implementation of urgent therapeutic measures.^145,146^

Most adverse events are mild and immediate, occur within the first infusions, are related to the rate of infusion, and are rapidly reversible.^115,145-152^

Headache, fever, malaise, flu-like symptoms, nausea, chills, fatigue, myalgia, low back pain, tachycardia, blood pressure changes and erythroderma are the most common events.^115,144,146,147,149,152^

Severe reactions occur in less than 1% of infusions and usually with higher doses indicated for autoimmune and inflammatory diseases.^75,144,152,153^

The exact pathophysiology of adverse events is still unknown. Some possibilities have been raised over the years, such as formation of IgG aggregates, interaction between the infused IgG and microbial antigens circulating in patients leading to formation of immune complexes, and reaction to vasoactive plasma components, contaminants, or other ingredients used during processing.^115,116,144,147,149^

Anaphylactic-type reactions mostly do not involve IgE. They usually evolve with hypertension instead of hypotension, and are less severe in subsequent infusions.^116,144,151^ IgE-mediated anaphylaxis is a very rare event in patients with absence of IgA and preserved IgE production.^144^ In these cases, the use of low-IgA intravenous preparations or subcutaneous Ig infusions is indicated.^16,116,154^ However, there is no need to assess the presence of anti-IgA antibodies before starting Ig therapy.^144,154^

Although rare, there are descriptions of neurological, respiratory, cardiovascular, gastrointestinal, renal, skin and blood abnormalities including headaches, aseptic meningitis, dyspnea, bronchospasm, transfusion-related acute lung injury (TRALI)*,* hypotension or hypertension, arrhythmias, nausea, vomiting, diarrhea, renal failure, hives, skin rash, pruritic dermatosis, hemolytic anemia and thromboembolic events (Chart 3).^16,75,115,144,147,149,151,153,155^

**Chart 3:** Types and frequency of adverse effects associated with administration of intravenous immunoglobulin

Symptoms and signs	Frequency
Related to infusion rate	
Chills	Frequent
Headache	Frequent
Dyspnea	Frequent
Chest pain or tightness	Frequent
Back pain	Frequent
Fatigue and malaise	Frequent
Fever	Frequent
Hypotension or hypertension	Frequent
Myalgia	Frequent
Nausea and vomiting	Frequent
Pruritus	Frequent
Skin rash and hives	Frequent
Flu-like symptoms	Frequent
Tachycardia	Frequent
Central nervous system	
Aseptic meningitis	Rare
Severe headache	Rare
Renal	
Acute renal failure (acute tubular necrosis)	Rare (usually associated with sucrose as a stabilizer)
Azotemia	Rare
Thromboembolic events	
Thrombosis and cerebral infarction	Rare
Myocardial infarction	Rare
Pulmonary thromboembolism	Rare
Posterior leukoencephalopathy syndrome	Rare
Other	
Anti-IgA IgE-mediated anaphylaxis	Very rare
Abnormal heart rhythm	Isolated reports (very rare)
Coagulopathy	Isolated reports (very rare)
Hemolysis – alloantibodies against A and B blood types	Isolated reports (very rare)
Cryoglobulinemia	Isolated reports (very rare)
Neutropenia	Isolated reports (very rare)
Alopecia	Isolated reports (very rare)
Uveitis	Isolated reports (very rare)
Non-infectious hepatitis	Isolated reports (very rare)

Source: Ballow MC. Immunoglobulin therapy: replacement and immunomodulation. In: Rich RR, editor. Clinical immunology: principles and practice. 4th. USA: Elsevier; 2013. p. 1041-63;^(60)^ Späth PJ, Granata G, La Marra F, Kuijpers TW, Quinti I. On the dark side of therapies with immunoglobulin concentrates: the adverse events. Front Immunol. 2015;6:11. Review.^(115)^

IgA: immunoglobulin A; IgE: immunoglobulin E.

Some factors are associated with a higher risk of adverse effects and are listed in chart 4.^1,41,47,59,116,144,146,148,151-153,155,156^ It is worth noting that the presence of adverse events varies between different products, or even between different batches of the same product. Some patients have adverse effects with one or more Ig products, but not all of them.^144^

**Chart 4:** Factors associated with a greater rate of adverse effects of intravenous immunoglobulin

Presence of infections
Fever with no apparent source
Dehydration
Obesity
Age over 65 years
High blood pressure, heart disease or kidney disease
Concomitant use of nephrotoxic drugs
Hypercoagulable states
First infusions
Long interval between infusions
Product switching
Products with high concentrations (and high osmolarity)
Products with high sodium and/or sugar content
High rate of infusion
Higher doses

Considering the predisposing factors presented, proper measures must be taken to prevent adverse effects resulting from intravenous Ig infusions (Chart 5).^1,47,116,146,148,151,157^

**Chart 5:** Measures to prevent adverse effects of intravenous immunoglobulin

Control of predisposing factors: treat infectious processes and slow down infusion in case of major infection, avoid product switching, avoid long periods between infusions
Pre-hydration (30 minutes prior) with 0.9% saline solution, 10 to 20mL/kg in children, and 500mL in adults
Allow product to reach room temperature
Properly reconstitute lyophilized products
Monitor vital signs every 20 to 30 minutes
Slow infusion rate, particularly in first infusions, and using infusion pumps, whenever possible. Start at 0.01mL/kg/minute (0.5 to 1mg/kg/minute), increasing gradually (every 15 to 30 minutes) to 0.02mL/kg/min, 0.04mL/kg/min, 0.06mL/kg/min up to 0.08mL/kg/min (4 to 8mg/kg/min, respectively for products at 5 and 10%), over 3 to 6 hours
A scaled regimen with shorter intervals can be used in subsequent infusions, or even continuous infusion, as tolerated by the patient
Observe for 30 to 60 minutes after completion, before releasing the patient

Most adverse effects can be resolved by reducing the rate or briefly stopping the infusion, and by giving analgesics and/or anti-histamines.^5,146,147,151^ Some patients may require corticosteroids.^146,149,158^

In case of adverse reactions during intravenous administration, proper measures must be taken for future infusions (Chart 6).^146,147,151,157^

**Chart 6:** Measures for secondary prevention of adverse reactions to intravenous immunoglobulin

Slower rate of infusion in patients with prior reaction
Pre-medication with analgesics and/or nonsteroidal anti-inflammatory drugs, H1 (and anti H2) antihistamines, and corticosteroids
Pre-hydration with 0.9% saline solution
Switch product or consider subcutaneous Ig in case of major reactions with no response to symptomatic drugs

Ig: immunoglobulin.

Special attention is needed for patients with comorbidities, such as heart diseases, kidney diseases, liver diseases, coagulation disorders (thrombophilia), and *diabetes mellitus*. In these situations, some product characteristics, such as the presence of sugars, osmolality, sodium, among others, must be assessed. Chart 7 describes the most relevant factors according to the associated morbidity, and chart 8 lists the products available to facilitate this choice.^5,117,147^

**Chart 7:** Immunoglobulin characteristics to be assessed before prescribing commercial intravenous immunoglobulin products, considering comorbidities and age groups

Comorbidities and age groups	Characteristics of Ig products						

	Volume	Osmolarity	Sodium	Sugar	Other stabilizers	pH	IgA
Heart failure	x	x	x	-	x Glycine	x	-
Renal failure	x	x	x	x Sucrose-glucose	-	-	-
Anti-IgA antibodies	-	-	-	-	-	-	x
Thromboembolic risk	x	x	x	-	-	-	-
*Diabetes mellitus*	-	-	-	x Glucose-maltose	-	-	-
Hyperprolinemia	-	-	-	-	x L-proline	-	-
Hereditary fructose intolerance	-	-	-	x Sorbitol	-	-	-
Corn allergy	-	-	-	x Maltose	-	-	-
Seniors	x	x	x	x Glucose	-	-	-
							
Newborn/children	x	x	x	-	-	x	-

Source: modified from Abolhassani H, Asgardoon MH, Rezaei N, Hammarstrom L, Aghamohammadi A. Different brands of intravenous immunoglobulin for primary immunodeficiencies: how to choose the best option for the patient? Expert Rev Clin Immunol. 2015;11(11):1229-43. Review.^(5)^

IgA: immunoglobulin A.

**Chart 8:** Human immunoglobulin, commercial products available in Brazil

Brand name	Manufacturer/distributor	Sugar	Sodium	Osmolarity	pH	IgA concentration
Intravenous use						
Endobulin Kiovig 10% solution	Baxter Hospitalar	Does not contain	Does not contain	240-300mOsmoI/kg	46-5.1	Maximum: 0.14mg/mL
Flebogamma DIF 5% solution	Grifols	D-Sorbitol	<3.2mmol/L	32±4.5mOsmI/kg	5.6±0.1	<0.003mg/mL
Flebogamma DIF 10% solution	Grifols	D-Sorbitol	<3.2mmol/L	342±7.2mOsmI/kg	5.5±0.1	<0.003mg/mL
Blau* Immunoglobulin	Blausiegel	Maltose	-	-	-	-
OCTAGAM^®^ 5% solution	Octapharma	Maltose	≤0.015mmol/mL	310-380mOsmol/Kg	5.1-6.0	<0.2mg/mL
OCTAGAM^®^ 10% solution	Octapharma	Maltose	≤0.03mmol/mL	≥240mOsmol/Kg	4.5-5.0	<0.4mg/mL
Privigen	CSL Behring	Does not contain	Does not contain	320mOsmol/kg	4.8	≤0.025g/L
TEGELINE^®^ 5% lyophilized powder	LFB	Sucrose	2mg/mL NaCL	340-480mOsmol/kg	4.0-7.4	Maximum: 17mg/g
TEGELINE^®^ NEWY 5% solution	LFB	Mannitol	Does not contain	270-330mOsmol/kg	4.0-7.4	Maximum: 0.022mg/mL
Vigam^®^*	Meizler	Sucrose	<160mmol/L	>240mOsmol/kg	-	<100mcg/mL
Subcutaneous use						
Endobulin Kiovig 10% solution†	Baxter Hospitalar	Does not contain	Does not contain	240-300mOsmol/kg	4.6-5.1	Maximum: 0.14mg/mL
Hizentra^®‡^	CSL™ Behring	Does not contain	Does not contain	380mOsmol/kg	4.8	Maximum content: 50mcg/L

Source: data obtained from manufacturers.

* Data obtained from product inserts; ^†^ Product previously approved only for intravenous use, recently released for subcutaneous use (Resolution 1789 of June 19, 2015, published in the Official Federal Gazette of June 22, 2015); ^‡^ Product exclusively for subcutaneous use as recently approved by the National Health Surveillance Agency (ANVISA) (Resolution 2617 of September 18, 2015, published in the Official Federal Gazette of September 21, 2015).

Subcutaneous administration is rarely associated with systemic adverse effects, which occur in less than 1% of infusions.^42,72,144^ Approximately 75% of patients present with any grade of edema and hyperemia at the administration site which, however, usually resolve within 24 to 48 hours and tend to disappear in later infusions, without impairing treatment continuity using this route.^29,32,69,99,144,146,151,159,160^ Therefore, there is no need for pre-medications or medical supervision during infusions after proper training of patients/caregivers.^16,144^

It is important to report the adverse effects of Ig administration observed by physicians as well as patients and their caregivers, at the website NOTIVISA - Health Surveillance Reporting System: http://www.anvisa.gov.br/hotsite/notivisa/index.htm. The manufacturers, according to Decree 6523, of 31 July 2008, by the Chief of Staff, must have a customer service call center (SAC) with lines readily available. And physicians and patients must be able to use this call center to report signs and symptoms related with the use of different human Ig presentations commercially available.

## CHOOSING BETWEEN INTRAVENOUS AND SUBCUTANEOUS ADMINISTRATION

Treatment of patients with PID as well as other patients, particularly those with chronic diseases, must always be individualized to achieve good control of the disease and its manifestations, as well as good quality of life, and must be as adjusted as possible to patient characteristics and preferences.^27,41,70,116,123^

Each of the routes, intravenous or subcutaneous, has interesting features (Chart 9) depending on factors related to the disease, the patient and their family, as well as their socioeconomic level. What can be described as a disadvantage for a certain patient can be quite beneficial in other situations. For example, monthly intravenous application in a hospital could be interesting for patients with more severe disease, whose family does not adhere to the treatment, in which case close clinical monitoring is critical.

**Chart 9:** Comparison between intravenous and subcutaneous immunoglobulin

Items for comparison	Intravenous Ig	Subcutaneous Ig*
Infusion frequency	Every 3 to 4 weeks	From daily to every 2 weeks
Infusion volume	Large	Small
Infusion time	2 to 6 hours	30 to 90 minutes (pump) 5 to 20 minutes (push)
Use of high doses	Possible	Limited by volume/sites and number of sites
Control of serum IgG levels	Before each infusion	Anytime
Pharmacokinetics	Rapid rise in IgG levels after infusion, with subsequent fluctuating levels and wear-off effect	Slower increase in IgG levels, with subsequent stable levels and no wear-off effect
Infusion	Requires venous access secured by qualified professionals at a healthcare unit	No need for venous access, can be applied by the patient, caregiver or healthcare professional after training, can be administered at home
Efficacy	Effective in infection control	Effective in infection control
Infusion site reactions	Rare	Frequent but usually mild and improving with time
Systemic reactions	Rare, more prevalent in the first infusions and depending on the presence of comorbidities	Very rare
Level of patient satisfaction	Generally preferred by patients and caregivers who do not wish to self-administer or want less frequent applications	Overall improvement in the quality of life of patients who want independence and fewer trips to the healthcare unit, or patients who experience adverse events with intravenous Ig
Patient characteristics	Preferable in patients of low socioeconomic and education level requiring closer clinical follow-up, with poor adherence to the treatment, with extensive or severe skin lesions, coagulation disorders, and patients resistant to self-administration	Preferable in the presence of some comorbidities, difficult venous access, poor clinical control or significant adverse effects with intravenous infusion, difficult access to the healthcare facility; indicated for patients with good treatment adherence, good hygiene conditions at home, and trained and motivated to perform administration
Cost	Higher (product, healthcare facility, infusion supplies, healthcare staff)	Lower (product, infusion supplies and pump)

Source: Wasserman RL. Progress in gammaglobulin therapy for immunodeficiency: from subcutaneous to intravenous infusions and back again. J Clin Immunol. 2012;32(6):1153-64. Review;^(18)^ Kobrynski L. Subcutaneous immunoglobulin therapy: a new option for patients with primary immunodeficiency diseases. Biologics. 2012;6:277-87;^(35)^ Shapiro R. Subcutaneous immunoglobulin. Immunol Allergy Clin North Am. 2012. In press;^(36)^ Torgerson TR, Bonagura VR, Shapiro RS. Clinical ambiguities--ongoing questions. J Clin Immunol. 2013;33(Suppl 2):S99-103;^(41)^ Shabaninejad H, Asgharzadeh A, Rezaei N, Rezapoor A. A Comparative Study of Intravenous Immunoglobulin and Subcutaneous Immunoglobulin in Adult Patients with Primary Immunodeficiency Diseases: a systematic review and meta-analysis. Expert Rev Clin Immunol. 2016;12(5):595-602. Review;^(42)^ Peter JG, Chapel H. Immunoglobulin replacement therapy for primary immunodeficiencies. Immunotherapy. 2014;6(7):853-69. Review;^(48)^ Albin S, Cunningham-Rundles C. An update on the use of immunoglobulin for the treatment of immunode ciency disorders. Immunotherapy. 2014;6(10):1113-26. Review;^(78)^ Sriaroon P, Ballow M. Immunoglobulin Replacement Therapy for Primary Immunodeficiency. Immunol Allergy Clin North Am. 2015;35(4):713-30. Review;^(116)^ Berger M. Choices in IgG replacement therapy for primary immune deficiency diseases: subcutaneous IgG vs. intravenous IgG and selecting an optimal dose. Curr Opin Allergy Clin Immunol. 2011;11(6):532-8. Review;^(130)^ Abolhassani H, Sadaghiani MS, Aghamohammadi A, Ochs HD, Rezaei N. Home-based subcutaneous immunoglobulin versus hospital-based intravenous immunoglobulin in treatment of primary antibody deficiencies: systematic review and meta analysis. J Clin Immunol. 2012;32(6):1180-92. Review;^(161)^ Shapiro R. Why I use subcutaneous immunoglobulin (SCIG). J Clin Immunol. 2013;33 Suppl 2:S95-8. Review.^(173)^

* Considering the possibility of home infusion, not yet approved in Brazil.

Ig: immunoglobulin; IgG: immunoglobulin G.

The intravenous route allows for faster achievement of higher IgG concentrations, has documented efficacy and accommodates longer administration intervals. In addition, by infusing at the hospital, it is possible to have patients under stricter supervision by the healthcare staff. However, it is necessary to secure venous access, which can be difficult in many patients, and the procedure must be preferably performed at a hospital, requiring monthly visits. There is also systemic adverse effects, even if not frequent. The serum IgG concentrations obtained are unstable, with significant reduction 15 to 20 days after administration, sometimes associated with symptoms such as fatigue and malaise (wear-off effects).^48,130^

With subcutaneous use, serum IgG concentrations are more stable, allowing for easier application, without the need for venous access. In some countries, there is no need to visit the healthcare facility, offering greater independence to patients and caregivers.^48,161^ Other countries have vast experience with the subcutaneous route, with proven efficacy and safety,^31,35,104,160,162^ including fewer systemic adverse effects.^31,32,130,163^ Furthermore, there are many studies demonstrating improved quality of life with subcutaneous Ig replacement.^35,59,68,102,164^ Serum IgG concentrations rise more slowly, which could be considered a disadvantage in cases of very low initial IgG levels, but is an advantage in patients with hypersplenism or high renal/gastrointestinal loss.^165^ There is a need for training and engagement of patients and/or caregivers, which is usually possible to be achieved over 4 to 6 weeks, and close monitoring of the infusion technique must be maintained subsequently.^27,104,137,162^ There are several studies,^31,166-171^ including national studies,^172^ pointing to a considerable cost reduction associated with subcutaneous administration, particularly when performed at home.

Intravenous Ig is effective, safe, leads to a rapid rise in IgG concentrations, and can be obtained via the public healthcare system in Brazil (SUS - *Sistema Único de Saúde*) as well as the private system. Subcutaneous Ig has been offered only by the private healthcare system. The classic indications for subcutaneous Ig are problems with intravenous infusion: inadequate IgG concentrations, poor clinical control, wear-off, systemic adverse effects, difficulty securing venous access, or difficult access to healthcare facilities.^42,116,173^ Individual aspects which can improve the quality of life of patients must also be considered when this choice is made, as well as reduction of treatment costs.^18,27,41,57,71,116,173^

## MONITORING

Clinical and laboratory monitoring of patients must be performed to ensure good disease control and watch for complications and potential side effects of the therapy. It is critical to record the product brand, the lot number and the expiration date of every infusion.^52,60^

Regular clinical evaluations must be performed at variable intervals, depending on the severity of the PID, as well as personal, familial and social characteristics of the patient. It is important to observe the number, type and severity of infections, use of antibiotics, need for hospitalizations, attendance of every day activities (school or work), new complaints and symptoms, and presence of comorbidities, in addition to performing a complete physical examination.^5,27,59^

The following tests are recommended before the start of infusions: Ig levels (A, M, G and E), evaluation of vaccine response and lymphocyte count (T, B and NK), complete blood count, direct Coombs, kidney and liver function, and PCR for infectious agents (because these patients have impaired antibody production, serologic tests for infectious agents are not indicated).^47,57,116,144,157^

Laboratory control must be carried out every 3 to 6 months in the first year, and then every 6 to 12 months depending on the clinical condition. It must include^5,72,116,119^ serum IgG, and also IgA and IgM, particularly in very young patients, in order to detect recovery in patients with unspecified hypogammaglobulinemia; complete blood count; sedimentation rate; C-reactive protein; direct Coombs; and kidney and liver function tests.

## APPROVED PRODUCTS

The products approved for sales in Brazil are presented in chart 8.

The use of intravenous human Ig in antibody immunodeficiencies was regulated by the Clinical Protocol and Therapeutic Guidelines (PCDT) published in the SAS/MS Ordinance 495, of September 11, 2007, http://bvsms.saude.gov.br/bvs/saudelegis/sas/2007/ prt0495_11_09_2007.html. The document was subjected to public consultation nº. 22 of May 10, 2010, (http://bvsms.saude.gov.br/bvs/saudelegis/sas/2010/cop0022_10_05_2010.html) by the Health Care Department (Ministry of Health) with a proposal to update this PCDT which has not yet been published. The PDIs for which Ig therapy is indicated, according to this PCDT update proposal, are listed in chart 10.

**Chart 10:** Primary immunodeficiencies and International Classification of Diseases (ICD-10) for which immunoglobulin replacement is indicated, as per the 2010 proposal to update the Clinical Protocol and Therapeutic Guidelines for primary immunodeficiencies with antibody defects

D80.0 - Hereditary hypogammaglobulinemia
D80.1 - Non-familial hypogammaglobulinemia
D80.3 - Selective IgG subclass deficiency
D80.5 - Hyper IgM immunodeficiency
D80.6 - Antibody deficiency with near-normal immunoglobulins or hypergammaglobulinemia
D80.7 - Transient hypogammaglobulinemia of infancy*
D80.8 - Other immunodeficiencies with predominantly antibody defects
D81.0 - Severe combined immunodeficiency (SCID) with reticular dysgenesis
D81.1 - Severe combined immunodeficiency (SCID) with low T and B-cells
D81.2 - Severe combined immunodeficiency (SCID) with low or normal B-cells
D81.3 - Adenosine deaminase deficiency
D81.4 - Nezelof Syndrome
D81.5 - Purine nucleoside phosphorylase deficiency
D81.6 - Major histocompatibility complex class I deficiency
D81.7 - Major histocompatibility complex class II deficiency
D81.8 - Other combined immune deficiencies
D82.0 - Wiskott-Aldrich Syndrome
D82.1 - DiGeorge Syndrome
D83.0 - Common variable immunodeficiency with predominantly B-cell number and function abnormalities
D83.2 - Common variable immunodeficiency with anti-B or T-cell autoantibodies
D83.8 - Other common variable immunodeficiencies

Source: http://bvsms.saude.gov.br/bvs/saudelegis/sas/2010/cop0022_10_05_2010.html

## OBTAINING HUMAN IMMUNOGLOBULIN FOR THERAPEUTIC USE IN BRAZIL

Treatment with human Ig can be obtained via the private healthcare system (regulated by the National Health Agency - ANVISA) or the public system.

To obtain from the private healthcare system, the assisting physician must write a report with the rationale for use, clinical condition, results of diagnostic and auxiliary tests, containing the ICD-10 and the prescription of the product with the proposed dose and frequency, informing where infusions will be performed. Products for intravenous or subcutaneous use can be administered at infusion centers, hospitals, day-hospitals or at home for patients on home care.

Requests to the public healthcare system must be submitted to State health departments. Patients or caregivers must go to the address informed on the website of the competent health department with the original and copies of their National Health Card, identity card, taxpayer ID and proof of residence; application report, evaluation and authorization for specialized pharmaceutical assistance components (LME); medical report with rationale for use, clinical condition and diagnosis; results of auxiliary tests and two copies of the prescription; informed consent form. All information can be found on the websites of State Health Departments in Brazil.

The use of subcutaneous or intravenous Ig at home is not yet approved in our country, despite it being common in other countries,^27,29,37-39,41,161^ as well as demonstratedly effective and safe.^25,99,107,145,162,174^ A recent survey conducted by the International Patient Organisation for Primary Immunodeficiencies (IPOPI), in 20 countries, showed that among 300 patients, 53% received intravenous Ig and 45%, subcutaneous Ig, and that 14% of patients on intravenous Ig and 94% of patients on subcutaneous Ig received infusions at home.^138^

## CONCLUSION

Ever since the 1^st^ Brazilian Consensus on the Use of Human Immunoglobulin in Patients with Primary Immunodeficiencies, published in 2010, several new primary immunodeficiencies have been described. Since then, new human immunoglobulin products have been made available in our country, with different compositions and administration routes. Therefore, the recommendations for the use of immunoglobulin in our country need to be updated.

This work provides new knowledge on the products available, their indications, mode of use and monitoring information.

The indication of each product depends on clinical and laboratory characteristics of patients, and treatment individualization and patient monitoring are critical, irrespective of the brand or route of administration of the product.

## References

[1] Costa-Carvalho BT, Solé D, Condino A, Rosário N (2010). I Consenso Brasileiro sobre o Uso de Imunoglobulina Humana em Pacientes com Imunodeficiências Primárias. Rev Bras Alerg Imunopatol.

[2] Ochs HD, Hagin D (2014). Primary immunodeficiency disorders: general classification, new molecular insights, and practical approach to diagnosis and treatment. Ann Allergy Asthma Immunol.

[3] Rezaei N, Vries ED, Gambineri E, Haddad E, Sullivan KE, Stiehm ER (2014). Common presentations and diagnostic approaches. Stiehm’s Immune Deficiencies.

[4] Bousfiha A, Jeddane L, Al-Herz W, Ailal F, Casanova JL, Chatila T (2015). The 2015 IUIS Phenotypic Classification for Primary Immunodeficiencies. J Clin Immunol.

[5] Abolhassani H, Asgardoon MH, Rezaei N, Hammarstrom L, Aghamohammadi A (2015). Different brands of intravenous immunoglobulin for primary immunodeficiencies: how to choose the best option for the patient?. Expert Rev Clin Immunol.

[6] Picard C, Al-Herz W, Bousfiha A, Casanova JL, Chatila T, Conley ME (2015). Primary immunodeficiency diseases: an update on the classification from the nternational Union of Immunological Societies Expert Committee for Primary Immunodeficiency 2015. J Clin Immunol.

[7] Leiva LE, Zelazco M, Oleastro M, Carneiro-Sampaio M, Condino A, Costa-Carvalho BT, Grumach AS, Quezada A, Patiño P, Franco JL, Porras O, Rodríguez FJ, Espinosa-Rosales FJ, Espinosa-Padilla SE, Almillategui D, Martínez C, Tafur JR, Valentín M, Benarroch L, Barroso R, Sorensen RU, Latin American Group for Primary Immunodeficiency Diseases (2007). Primary immunodeficiency diseases in Latin America: the second report of the LAGID registry. J Clin Immunol.

[8] Ballow M, Notarangelo L, Grimbacher B, Cunningham-Rundles C, Stein M, Helbert M (2009). Immunodeficiencies. Clin Exp Immunol.

[9] Errante PR, Franco JL, Espinosa-Rosales FJ, Sorensen R, Condino A (2012). Advances in primary immunodeficiency diseases in Latin America: epidemiology, research, and perspectives. Ann N Y Acad Sci.

[10] Grimbacher B, ESID Registry Working Party (2014). The European Society for Immunodeficiencies (ESID) registry 2014. Clin Exp Immunol.

[11] Kobrynski L, Powell RW, Bowen S (2014). Prevalence and morbidity of primary immunodeficiency diseases, United States 2001-2007. J Clin Immunol.

[12] Mahlaoui N, Gathmann B, Kindle G, Ehl S, Quinti I, Grimbacher B (2014). The European Society for Immunodeficiencies (ESID) Registry: recent advances in the epidemiology of Primary Immunodeficiencies and how does that translate in clinical care. Rare Dis Orphan Drugs.

[13] Modell V, Quinn J, Orange J, Notarangelo LD, Modell F (2016). Primary immunodeficiencies worldwide: an updated overview from the Jeffrey Modell Centers Global Network. Immunol Res.

[14] Oncley J, Melin M, Richert D, Cameron J, Gross P (1949). The separation of the antibodies, isoagglutinins, prothrombin, plasminogen and beta1 lipoprotein into subfractions of human plasma. J Am Chem Soc.

[15] Eibl MM (2008). History of immunoglobulin replacement. Immunol Allergy Clin North Am.

[16] Berger M, Stiehm ER, Etzioni A, Ochs HD (2014). From subcutaneous to intravenous immunoglobulin and back. Primary immunodeficiency disorders: a historic and scientific perspective.

[17] Bruton O (1952). Agammaglobulinemia. Pediatrics.

[18] Wasserman RL (2012). Progress in gammaglobulin therapy for immunodeficiency: from subcutaneous to intravenous infusions and back again. J Clin Immunol.

[19] Wasserman RL, Stein M, Melamed I, Kobrynski LJ, Gupta S, Puck J (2015). Long-term efficacy and safety of recombinant human hyaluronidase (rHuPH20)- facilitated subcutaneous infusion of immunoglobulin G (IgG) (HyQvia; IGHy) in patients with primary immunodeficiencies (PI). J Allergy Clin Immunol.

[20] Skoda-Smith S, Torgerson T, Ochs HD (2010). Subcutaneous immunoglobulin replacement therapy in the treatment of patients with primary immunodeficiency disease. Ther Clin Risk Manag.

[21] Berger M, Cupps TR, Fauci AS (1980). Immunoglobulin replacement therapy by slow subcutaneous infusion. Ann Intern Med.

[22] Roord JJ, van der Meer JW, Kuis W, de Windt GE, Zegers BJ, van Furth R (1982). Home treatment in patients with antibody deficiency by slow subcutaneous infusion of gammaglobulin. Lancet.

[23] Ugazio AG, Duse M, Re R, Mangili G, Burgio GR (1982). Subcutaneous infusion of gammaglobulins in management of agammaglobulinaemia. Lancet.

[24] Roord JJ, van der Meer JW, Kuis W, van Furth R (1983). Treatment of antibody deficiency syndromes with subcutaneous infusion of gamma globulin. Birth Defects Orig Artic Ser.

[25] Chapel H, Brennan V, Delson E (1988). Immunoglobulin replacement therapy by self-infusion at home. Clin Exp Immunol.

[26] Gardulf A, Hammarström L, Smith CI (1991). Home treatment of hypogammaglobulinaemia with subcutaneous gammaglobulin by rapid infusion. Lancet.

[27] Chapel H, Gardulf A (2013). Subcutaneous immunoglobulin replacement therapy: the European experience. Curr Opin Allergy Clin Immunol.

[28] Gardulf A, Andersen V, Björkander J, Ericson D, Frøland SS, Gustafson R (1995). Subcutaneous immunoglobulin replacement in patients with primary antibody deficiencies: safety and costs. Lancet.

[29] Chapel HM, Spickett GP, Ericson D, Engl W, Eibl MM, Bjorkander J (2000). The comparison of the efficacy and safety of intravenous versus subcutaneous immunoglobulin replacement therapy. J Clin Immunol.

[30] Berger M (2004). Subcutaneous immunoglobulin replacement in primary immunodeficiencies. Clin Immunol.

[31] Gardulf A, Nicolay U, Asensio O, Bernatowska E, Böck A, Carvalho BC (2006). Rapid subcutaneous IgG replacement therapy is effective and safe in children and adults with primary immunodeficiencies--a prospective, multi-national study. J Clin Immunol.

[32] Gardulf A (2007). Immunoglobulin treatment for primary antibody deficiencies: advantages of the subcutaneous route. BioDrugs.

[33] Misbah S, Sturzenegger MH, Borte M, Shapiro RS, Wasserman RL, Berger M (2009). Subcutaneous immunoglobulin: opportunities and outlook. Clin Exp Immunol.

[34] Berger M, Murphy E, Riley P, Bergman GE, VIRTUE Trial Investigators (2010). Improved quality of life, immunoglobulin G levels, and infection rates in patients with primary immunodeficiency diseases during self-treatment with subcutaneous immunoglobulin G. South Med J.

[35] Kobrynski L (2012). Subcutaneous immunoglobulin therapy: a new option for patients with primary immunodeficiency diseases. Biologics.

[36] Shapiro R (2012). Subcutaneous immunoglobulin. Immunol Allergy Clin North Am.

[37] Bezrodnik L, Gómez Raccio A, Belardinelli G, Regairaz L, Diaz Ballve D, Seminario G (2013). Comparative study of subcutaneous versus intravenous IgG replacement therapy in pediatric patients with primary immunodeficiency diseases: a multicenter study in Argentina. J Clin Immunol.

[38] Bonagura VR (2013). Illustrative cases on individualizing immunoglobulin therapy in primary immunodeficiency disease. Ann Allergy Asthma Immunol.

[39] Gouilleux-Gruart V, Chapel H, Chevret S, Lucas M, Malphettes M, Fieschi C, Patel S, Boutboul D, Marson MN, Gérard L, Lee M, Watier H, Oksenhendler E (2013). Efficiency of immunoglobulin G replacement therapy in common variable immunodeficiency: correlations with clinical phenotype and polymorphism of the neonatal Fc receptor. Clin Exp Immunol.

[40] Lingman-Framme J, Fasth A (2013). Subcutaneous immunoglobulin for primary and secondary immunodeficiencies: an evidence-based review. Drugs.

[41] Torgerson TR, Bonagura VR, Shapiro RS (2013). Clinical ambiguities--ongoing questions. J Clin Immunol.

[42] Shabaninejad H, Asgharzadeh A, Rezaei N, Rezapoor A (2016). A Comparative Study of Intravenous Immunoglobulin and Subcutaneous Immunoglobulin in Adult Patients with Primary Immunodeficiency Diseases: a systematic review and meta-analysis. Expert Rev Clin Immunol.

[43] Sewell WA, Kerr J, Behr-Gross ME, Peter HH (2014). European consensus proposal for immunoglobulin therapies. Eur J Immunol.

[44] Bonilla FA, Bernstein IL, Khan DA, Ballas ZK, Chinen J, Frank MM, Kobrynski LJ, Levinson AI, Mazer B, Nelson RP, Orange JS, Routes JM, Shearer WT, Sorensen RU, American Academy of Allergy, Asthma and Immunology, American College of Allergy, Asthma and Immunology, Joint Council of Allergy, Asthma and Immunology (2005). Practice parameter for the diagnosis and management of primary immunodeficiency. Ann Allergy Asthma Immunol.

[45] Bonagura VR (2014). Dose and outcomes in primary immunodeficiency disorders. Clin Exp Immunol.

[46] Chapel H, Prevot J, Gaspar HB, Español T, Bonilla FA, Solis L, Drabwell J, Editorial Board for Working Party on Principles of Care at IPOPI (2014). Primary immune deficiencies - principles of care. Front Immunol.

[47] Condino A, Costa-Carvalho BT, Grumach AS, King A, Bezrodnik L, Oleastro M (2014). Guidelines for the use of human immunoglobulin therapy in patients with primary immunodeficiencies in Latin America. Allergol Immunopathol (Madr).

[48] Peter JG, Chapel H (2014). Immunoglobulin replacement therapy for primary immunodeficiencies. Immunotherapy.

[49] Roifman CM, Levison H, Gelfand EW (1987). High-dose versus low-dose intravenous immunoglobulin in hypogammaglobulinaemia and chronic lung disease. Lancet.

[50] Eijkhout HW, van Der Meer JW, Kallenberg CG, Weening RS, van Dissel JT, Sanders LA, Strengers PF, Nienhuis H, Schellekens PT (2001). The effect of two different dosages of intravenous immunoglobulin on the incidence of recurrent infections in patients with primary hypogammaglobulinemia: a randomized, double-blind, multicenter crossover trial. Ann Intern Med.

[51] Bonilla FA (2008). Pharmacokinetics of immunoglobulin administered via intravenous or subcutaneous routes. Immunol Allergy Clin North Am.

[52] Yong PL, Boyle J, Ballow M, Boyle M, Berger M, Bleesing J (2010). Use of intravenous immunoglobulin and adjunctive therapies in the treatment of primary immunodeficiencies: a working group report of and study by the Primary Immunodeficiency Committee of the American Academy of Allergy Asthma and Immunology. Clin Immunol.

[53] Rose ME, Lang DM (2006). Evaluating and managing hypogammaglobulinemia. Cleve Clin J Med.

[54] Roifman CM, Berger M, Notarangelo LD (2008). Management of primary antibody deficiency with replacement therapy: summary of guidelines. Immunol Allergy Clin North Am.

[55] Lucas M, Lee M, Lortan J, Lopez-Granados E, Misbah S, Chapel H (2010). Infection outcomes in patients with common variable immunodeficiency disorders: relationship to immunoglobulin therapy over 22 years. J Allergy Clin Immunol.

[56] Orange JS, Grossman WJ, Navickis RJ, Wilkes MM (2010). Impact of trough IgG on pneumonia incidence in primary immunodeficiency: a meta-analysis of clinical studies. Clin Immunol.

[57] Shehata N, Palda V, Bowen T, Haddad E, Issekutz TB, Mazer B (2010). The use of immunoglobulin therapy for patients with primary immune deficiency: an evidence-based practice guideline. Transfus Med Rev.

[58] Ballow M (2012). Intravenous IgG replacement in PIDD: dose and clinical expectations. Immunol Allergy Clin North Am.

[59] Orange JS, Belohradsky BH, Berger M, Borte M, Hagan J, Jolles S (2012). Evaluation of correlation between dose and clinical outcomes in subcutaneous immunoglobulin replacement therapy. Clin Exp Immunol.

[60] Ballow MC, Rich RR (2013). Immunoglobulin therapy: replacement and immunomodulation. Clinical immunology: principles and practice.

[61] Roifman CM, Gelfand EW (1988). Replacement therapy with high dose intravenous gamma-globulin improves chronic sinopulmonary disease in patients with hipogammaglobulinemia. Pediatr Infect Dis J.

[62] Kainulainen L, Varpula M, Liippo K, Svedström E, Nikoskelainen J, Ruuskanen O (1999). Pulmonary abnormalities in patients with primary hypogammaglobulinemia. J Allergy Clin Immunol.

[63] Notarangelo LD, Plebani A, Mazzolari E, Soresina A, Bondioni MP (2007). Genetic causes of bronchiectasis: primary immune deficiencies and the lung. Respiration.

[64] Rich AL, Le Jeune IR, McDermott L, Kinnear WJ (2008). Serial lung function tests in primary immune deficiency. Clin Exp Immunol.

[65] Bonagura VR, Marchlewski R, Cox A, Rosenthal DW (2008). Biologic IgG level in primary immunodeficiency disease: the IgG level that protects against recurrent infection. J Allergy Clin Immunol.

[66] Bonagura VR (2013). Using intravenous immunoglobulin (IVIG) to treat patients with primary immune deficiency disease. J Clin Immunol.

[67] Jolles S, Orange JS, Gardulf A, Stein MR, Shapiro R, Borte M (2015). Current treatment options with immunoglobulin G for the individualization of care in patients with primary immunodeficiency disease. Clin Exp Immunol.

[68] Gardulf A, Nicolay U, Math D, Asensio O, Bernatowska E, Böck A (2004). Children and adults with primary antibody deficiencies gain quality of life by subcutaneous IgG self-infusions at home. J Allergy Clin Immunol.

[69] Berger M (2008). Subcutaneous administration of IgG. Immunol Allergy Clin North Am.

[70] American Academy of Allergy Asthma & Immunology (2011). Eight Guiding Principles for Effective Use of IVIG for Patients with Primary Immunodeficiency.

[71] Soler-Palacín P, Gasó-Gago I, Fernández-Polo A, Martín-Nalda A, Oliveras M, Martinez-Cutillas J (2014). Intravenous and subcutaneous immunoglobulin replacement: a two-way road. Optimizing healthcare quality in patients with primary immunodeficiencies. J Clin Immunol.

[72] Berger M (2008). Principles of and advances in immunoglobulin replacement therapy for primary immunodeficiency. Immunol Allergy Clin North Am.

[73] European Primary Immunodeficiencies Consensus Conference (2006). Consensus Report and Recommendations.

[74] Clark P, Wimperis J, Lunn M, Jones A, Herriot R, Wood D (2012). Clinical Guidelines for Immunoglobulin Use.

[75] Orange JS, Hossny EM, Weiler CR, Ballow M, Berger M, Bonilla FA, Buckley R, Chinen J, El-Gamal Y, Mazer BD, Nelson RP, Patel DD, Secord E, Sorensen RU, Wasserman RL, Cunningham-Rundles C, Primary Immunodeficiency Committee of the American Academy of Allergy, Asthma and Immunology (2006). Use of intravenous immunoglobulin in human disease: a review of evidence by members of the Primary Immunodeficiency Committee of the American Academy of Allergy, Asthma and Immunology. J Allergy Clin Immunol.

[76] Stiehm ER, Orange JS, Ballow M, Lehman H (2010). Therapeutic use of immunoglobulins. Adv Pediatr.

[77] Orange JS, Ochs HD, Cunningham-Rundles C (2013). Prioritization of evidence-based indications for intravenous immunoglobulin. J Clin Immunol.

[78] Albin S, Cunningham-Rundles C (2014). An update on the use of immunoglobulin for the treatment of immunode ciency disorders. Immunotherapy.

[79] Bonilla FA, Khan DA, Ballas ZK, Chinen J, Frank MM, Hsu JT, Keller M, Kobrynski LJ, Komarow HD, Mazer B, Nelson RP, Orange JS, Routes JM, Shearer WT, Sorensen RU, Verbsky JW, Bernstein DI, Blessing-Moore J, Lang D, Nicklas RA, Oppenheimer J, Portnoy JM, Randolph CR, Schuller D, Spector SL, Tilles S, Wallace D (2015). Practice parameter for the diagnosis and management of primary immunodeficiency. J Allergy Clin Immunol.

[80] Kerr J, Quinti I, Eibl M, Chapel H, Späth PJ, Sewell WA (2014). Is dosing of therapeutic immunoglobulins optimal? A review of a three-decade long debate in europe. Front Immunol.

[81] Matucci A, Maggi E, Vultaggio A (2015). Mechanisms of action of Ig preparations: immunomodulatory and anti-inflammatory effects. Front Immunol.

[82] Grimbacher B, Schäffer AA, Peter HH (2004). The genetics of hypogammaglobulinemia. Curr Allergy Asthma Rep.

[83] Garcia-Lloret M, McGhee S, Chatila TA (2008). Immunoglobulin replacement therapy in children. Immunol Allergy Clin North Am.

[84] Dhalla F, Misbah SA (2015). Secondary antibody deficiencies. Curr Opin Allergy Clin Immunol.

[85] Compagno N, Malipiero G, Cinetto F, Agostini C (2014). Immunoglobulin replacement therapy in secondary hypogammaglobulinemia. Front Immunol.

[86] Casulo C, Maragulia J, Zelenetz AD (2013). Incidence of hypogammaglobulinemia in patients receiving rituximab and the use of intravenous immunoglobulin for recurrent infections. Clin Lymphoma Myeloma Leuk.

[87] Compagno N, Cinetto F, Semenzato G, Agostini C (2014). Subcutaneous immunoglobulin in lymphoproliferative disorders and rituximab-related secondary hypogammaglobulinemia: a single-center experience in 61 patients. Haematologica.

[88] Aguiar R, Araújo C, Martins-Coelho G, Isenberg D (2017). Use of Rituximab in systemic lupus erythematosus: a single center experience over 14 Years. Arthritis Care Res (Hoboken).

[89] Fujinaga S, Ozawa K, Sakuraya K, Yamada A, Shimizu T (2016). Late-onset adverse events after a single dose of rituximab in children with complicated steroid-dependent nephrotic syndrome. Clin Nephrol.

[90] Spadaro G, Pecoraro A, De Renzo A, Della Pepa R, Genovese A (2016). Intravenous versus subcutaneous immunoglobulin replacement in secondary hypogammaglobulinemia. Clin Immunol.

[91] Nishio M, Endo T, Fujimoto K, Yamamoto S, Obara M, Yamaguchi K (2009). FCGR3A-158V/F polymorphism may correlate with the levels of immunoglobulin in patients with non-Hodgkin’s lymphoma after rituximab treatment as an adjuvant to autologous stem cell transplantation. Eur J Haematol.

[92] Kaplan B, Kopyltsova Y, Khokhar A, Lam F, Bonagura V (2014). Rituximab and immune deficiency: case series and review of the literature. J Allergy Clin Immunol Pract.

[93] Ammann AJ, Ashman RF, Buckley RH, Hardie WR, Krantmann HJ, Nelson J (1982). Use of intravenous gama-globulin in antibody immunodeficiency: results of a multicenter trial. Clin Immunol Immunopathol.

[94] Roifman CM, Lederman HM, Lavi S, Stein LD, Levison H, Gelfand EW (1985). Benefit of intravenous IgG replacement in hypogammaglobulinemic patients with chronic sinopulmonary disease. Am J Med.

[95] Buckley RH, Schiff RI (1991). The use of intravenous immunoglobulin in immunodeficiency diseases. N Engl J Med.

[96] Busse PJ, Razvi S, Cunningham-Rundles C (2002). Efficacy of intravenous immunoglobulin in the prevention of pneumonia in patients with common variable immunodeficiency. J Allergy Clin Immunol.

[97] Maarschalk-Ellerbroek LJ, Hoepelman IM, Ellerbroek PM (2011). Immunoglobulin treatment in primary antibody deficiency. Int J Antimicrob Agents.

[98] Gardulf A, Björvell H, Gustafson R, Hammarström L, Smith CI (1993). The life situations of patients with primary antibody deficiency untreated or treated with subcutaneous gammaglobulin infusions. Clin Exp Immunol.

[99] Ochs HD, Gupta S, Kiessling P, Nicolay U, Berger M, Subcutaneous IgG Study Group (2006). Safety and efficacy of self-administered subcutaneous immunoglobulin in patients with primary immunodeficiency diseases. J Clin Immunol.

[100] Hoffmann F, Grimbacher B, Thiel J, Peter HH, Belohradsky BH (2010). Home-based subcutaneous immunoglobulin G replacement therapy under real-life conditions in children and adults with antibody deficiency. Eur J Med Res.

[101] Gregory R, Malcolmson C, Patel C, Woolley T, Jones A (2013). Experience with a 20% subcutaneous immunoglobulin (Hizentra^®^) in children with primary immunodeficiency diseases - a single-center review. J Allergy Clin Immunol.

[102] Bourdin A, Berger J, Früh A, Spertini F, Bugnon O (2015). Subcutaneous immunoglobulin and support program: what level of interest of patients?. Rev Med Suisse.

[103] Jolles S (2016). Subcutaneous and intramuscular immune globulin therapy.

[104] Vultaggio A, Azzari C, Milito C, Finocchi A, Toppino C, Spadaro G (2015). Subcutaneous immunoglobulin replacement therapy in patients with primary immunodeficiency in routine clinical practice: the VISPO prospective multicenter study. Clin Drug Investig.

[105] Thomas MJ, Brennan VM, Chapel HH (1993). Rapid subcutaneous immunoglobulin infusions in children. Lancet.

[106] Gaspar J, Gerritsen B, Jones A (1998). Immunoglobulin replacement treatment by rapid subcutaneous infusion. Arch Dis Child.

[107] Fasth A, Nyström J (2007). Safety and efficacy of subcutaneous human immunoglobulin in children with primary immunodeficiency. Acta Paediatr.

[108] Church JA, Howard V, Sleasman JW, Borte M, Berger M (2011). Subcutaneous Immunoglobulin Replacement Therapy in Infants and Children with Primary Immunodeficiencies. J Allergy Clin Immunol.

[109] Shapiro RS (2013). Subcutaneous immunoglobulin: rapid push vs. infusion pump in pediatrics. Pediatric Allergy Immunol.

[110] Stein MR, Koterba A, Rodden L, Berger M (2011). Safety and efficacy of home-based subcutaneous immunoglobulin G in elderly patients with primary immunodeficiency diseases. Postgrad Med.

[111] Gardulf A, Andersson E, Lindqvist M, Hansen S, Gustafson R (2001). Rapid subcutaneous IgG replacement therapy at home for pregnant immunodeficient women. J Clin Immunol.

[112] Shapiro R (2013). Subcutaneous immunoglobulin (16 or 20%) therapy in obese patients with primary immunodeficiency: a retrospective analysis of administration by infusion pump or subcutaneous rapid push. Clin Exp Immunol.

[113] Hodkinson JP, Lucas M, Lee M, Harrison M, Lunn MP, Chapel H (2015). Therapeutic immunoglobulin should be dosed by clinical outcome rather than by body weight in obese patients. Clin Exp Immunol.

[114] Hooper JA (2008). Intravenous immunoglobulins: evolution of commercial IVIG preparations. Immunol Allergy Clin North Am.

[115] Späth PJ, Granata G, La Marra F, Kuijpers TW, Quinti I (2015). On the dark side of therapies with immunoglobulin concentrates: the adverse events. Front Immunol.

[116] Sriaroon P, Ballow M (2015). Immunoglobulin Replacement Therapy for Primary Immunodeficiency. Immunol Allergy Clin North Am.

[117] Saeedian M, Randhawa I (2014). Immunoglobulin replacement therapy: a twenty-year review and current update. Int Arch Allergy Immunol.

[118] International Patient Organisation for Primary Immunodeficiencies (IPOPI) (2012). The global organisation working to improve the quality of life for people with primary immunodeficiencies. Access to Immunoglobulin Therapies for patients living with a Primary Immunodeficiency.

[119] Orange JS (2015). Immune globulin therapy in primary immunodeficiency.

[120] Koleba T, Ensom MH (2006). Pharmacokinetics of intravenous immunoglobulin: a systematic review. Pharmacotherapy.

[121] Stiehm ER (1997). Human intravenous immunoglobulin in primary and secondary antibody deficiencies. Pediatr Infect Dis J.

[122] Andresen I, Kovarik JM, Psycher M, Bolli R (2000). Product equivalence study comparing the tolerability, pharmacokinetics, and pharmacodynamics of various human immunoglobulin-G Formulations. J Clin Pharmacol.

[123] Hernandez-Trujillo HS, Chapel H, Lo Re V, Notarangelo LD, Gathmann B, Grimbacher B (2012). Comparison of American and European practices in the management of patients with primary immunodeficiencies. Clin Exp Immunol.

[124] Wasserman RL, Melamed I, Nelson RP, Knutsen AP, Fasano MB, Stein MR (2011). Pharmacokinetics of subcutaneous IgPro20 in patients with primary immunodeficiency. Clin Pharmacokinet.

[125] Waniewski J, Gardulf A, Hammarstrom L (1994). Bioavailability of gamma-globulin after subcutaneous infusions in patients with common variable immunodeficiency. J Clin Immunol.

[126] Borte M, Quinti I, Soresina A, Fernández-Cruz E, Ritchie B, Schmidt DS (2011). Efficacy and safety of subcutaneous vivaglobin^(R)^ replacement therapy in previously untreated patients with primary immunodeficiency: a prospective, multicenter study. J Clin Immunol.

[127] Rojavin M, Sidhu J, Pfister M, Hubsch A (2014). Subcutaneous immunoglobulin loading regimens for previously untreated patients with primary antibody deficiency. Clin Exp Immunol.

[128] Koterba AP, Farnan K, Sierra C, Eufrasio D, Stein M (2012). Experience with subcutaneous loading of vivaglobin or hizentra in primary immunodeficiency patients naive to immunoglobulin replacement therapy. Ann Allergy Asthma Immunol.

[129] Koterba AP, Stein MR (2014). Initiation of immunoglobulin therapy by subcutaneous administration in immunodeficiency patients naive to replacement therapy. Allergy Asthma Clin Immunol.

[130] Berger M (2011). Choices in IgG replacement therapy for primary immune deficiency diseases: subcutaneous IgG vs. intravenous IgG and selecting an optimal dose. Curr Opin Allergy Clin Immunol.

[131] Berger M, Rojavin M, Kiessling P, Zenker O (2011). Pharmacokinetics of subcutaneous immunoglobulin and their use in dosing of replacement therapy in patients with primary immunodeficiencies. Clin Immunol.

[132] Younger ME, Blouin W, Duff C, Epland KB, Murphy E, Sedlak D (2015). Subcutaneous immunoglobulin replacement therapy: ensuring success. J Infus Nurs.

[133] Gustafson R, Gardulf A, Hansen S, Leibl H, Engl W, Lindén M (2008). Rapid subcutaneous immunoglobulin administration every second week results in high and stable serum immunoglobulin G levels in patients with primary antibody deficiencies. Clin Exp Immunol.

[134] Shapiro R (2010). Subcutaneous immunoglobulin therapy by rapid push is preferred to infusion by pump: a retrospective analysis. J Clin Immunol.

[135] Shapiro RS (2013). Subcutaneous immunoglobulin therapy given by subcutaneous rapid push vs infusion pump: a retrospective analysis. Ann Allergy Asthma Immunol.

[136] Younger ME, Aro L, Blouin W, Duff C, Epland KB, Murphy E, Sedlak D, Nurse Advisory Committee Immune Deficiency Foundation (2013). Nursing guidelines for administration of immunoglobulin replacement therapy. J Infus Nurs.

[137] Jolles S, Sleasman JW (2011). Subcutaneous immunoglobulin replacement therapy with Hizentra, the first 20% SCIG preparation: a practical approach. Adv Ther.

[138] International Patient Organisation for Primary Immunodeficiencies (IPOPI) (2012). Primary immunodeficiencies. Patient Needs & Outlooks Survey: a Report based on 300 patient questionnaires.

[139] Stein MR (2012). Enzyme-facilitated subcutaneous IgG: changing the paradigm of IgG therapy. Immunol Allergy Clin North Am.

[140] Ponsford M, Carne E, Kingdon C, Joyce C, Price C, Williams C (2015). Facilitated subcutaneous immunoglobulin (fSCIg) therapy--practical considerations. Clin Exp Immunol.

[141] Smith CI, Hammarström L (1985). Intravenous immunoglobulin in pregnancy. Obstet Gynecol.

[142] Williams PE, Leen CL, Heppleston AD, Yap PL (1990). IgG replacement therapy for primary hypogammaglobulinaemia during pregnancy: report of 9 pregnancies in 4 patients. Blut.

[143] Memmedova L, Azarsiz E, Edeer Karaca N, Aksu G, Kutukculer N (2013). Does intravenous immunoglobulin therapy prolong immunodeficiency in transient hypogammaglobulinemia of infancy?. Pediatr Rep.

[144] Stiehm ER (2013). Adverse effects of human immunoglobulin therapy. Transfus Med Rev.

[145] Brennan VM, Salomé-Bentley NJ, Chapel HM, Immunology Nurses Study (2003). Prospective audit of adverse reactions occurring in 459 primary antibody-deficient patients receiving intravenous immunoglobulin. Clin Exp Immunol.

[146] Bonilla FA (2008). Intravenous immunoglobulin: adverse reactions and management. J Allergy Clin Immunol.

[147] Cherin P, Marie I, Michallet M, Pelus E, Dantal J, Crave JC (2016). Management of adverse events in the treatment of patients with immunoglobulin therapy: a review of evidence. Autoimmun Rev.

[148] Katz U, Achiron A, Sherer Y, Shoenfeld Y (2007). Safety of intravenous immunoglobulin (IVIG) therapy. Autoimmun Rev.

[149] Ballow M (2007). Safety of IGIV therapy and infusion-related adverse events. Immunol Res.

[150] Dashti-Khavidaki S, Aghamohammadi A, Farshadi F, Movahedi M, Parvaneh N, Pouladi N (2009). Adverse reactions of prophylactic intravenous immunoglobulin; a 13-year experience with 3004 infusions in iranian patients with primary immunodeficiency diseases. J Investig Allergol Clin Immunol.

[151] Berger M (2013). Adverse effects of IgG therapy. J Allergy Clin Immunol Pract.

[152] Bichuetti-Silva DC, Furlan FP, Nobre FA, Pereira CT, Goncalves TR, Gouveia-Pereira M (2014). Immediate infusion-related adverse reactions to intravenous immunoglobulin in a prospective cohort of 1765 infusions. Int Immunopharmacol.

[153] Ramírez E, Romero-Garrido JA, López-Granados E, Borobia AM, Pérez T, Medrano N (2014). Symptomatic thromboembolic events in patients treated with intravenous-immunoglobulins: results from a retrospective cohort study. Thromb Res.

[154] Rachid R, Bonilla FA (2012). The role of anti-IgA antibodies in causing adverse reactions to gamma globulin infusion in immunodeficient patients: a comprehensive review of the literature. J Allergy Clin Immunol.

[155] Gelfand EW (2012). Intravenous immune globulin in autoimmune and inflammatory diseases. N Engl J Med.

[156] Bonilla FA (2014). Adverse effects of immunoglobulin G therapy: thromboembolism and haemolysis. Clin Exp Immunol.

[157] Silvergleid AJ, Ballow M, Schrier SL, Stiehm ER, Tirnauer JS (2016). Overview of intravenous immune globulin (IVIG) therapy.

[158] Singh-Grewal D, Kemp A, Wong M (2006). A prospective study of the immediate and delayed adverse events following intravenous immunoglobulin infusions. Arch Dis Child.

[159] Melamed I, Testori A, Spirer Z (2012). Subcutaneous immunoglobulins: product characteristics and their role in primary immunodeficiency disease. Int Rev Immunol.

[160] Karakoç Aydıner E, Kıykım A, Barış S, Özen A, Barlan I (2016). Use of subcutaneous immunoglobulin in primary immune deficiencies. Turk Pediatri Ars.

[161] Abolhassani H, Sadaghiani MS, Aghamohammadi A, Ochs HD, Rezaei N (2012). Home-based subcutaneous immunoglobulin versus hospital-based intravenous immunoglobulin in treatment of primary antibody deficiencies: systematic review and meta analysis. J Clin Immunol.

[162] Bhole MV, Burton J, Chapel HM (2008). Self-infusion programmes for immunoglobulin replacement at home: feasibility, safety and efficacy. Immunol Allergy Clin North Am.

[163] Markvardsen LH, Christiansen. I, Andersen H, Jakodsen J (2015). Headache and nausea after treatment with high-dose subcutaneous versus intravenous immunoglobulin. Basic Clin Pharmacol Toxicol.

[164] Nicolay U, Kiessling P, Berger M, Gupta S, Yel L, Roifman CM (2006). Health-related quality of life and treatment satisfaction in North American patients with primary immunedeficiency diseases receiving subcutaneous IgG self-infusions at home. J Clin Immunol.

[165] Shah SN, Todoric K, Tarrant TK (2015). Improved outcomes on subcutaneous IgG in patients with humoral immunodeficiency and co-morbid bowel disease. Clin Case Rep Rev.

[166] Högy B, Keinecke HO, Borte M (2005). Pharmacoeconomic evaluation of immunoglobulin treatment in patients with antibody deficiencies from the perspective of the German statutory health insurance. Eur J Health Econ.

[167] Haddad L, Perrinet M, Parent D, Leroy-Cotteau A, Toguyeni E, Condette-Wojtasik G (2006). Economic evaluation of at home subcutaneous and intravenous immunoglobulin substitution. Rev Med Interne.

[168] Beauté J, Levy P, Millet V, Debré M, Dudoit Y, Le Mignot L, Tajahmady A, Thomas C, Suarez F, Pellier I, Hermine O, Aladjidi N, Mahlaoui N, Fischer A, French PID study group CEREDIH (2010). Economic evaluation of immunoglobulin replacement in patients with primary antibody deficiencies. Clin Exp Immunol.

[169] Ducruet T, Levasseur MC, Des Roches A, Kafal A, Dicaire R, Haddad E (2013). Pharmacoeconomic advantages of subcutaneous versus intravenous immunoglobulin treatment in a Canadian pediatric center. J Allergy Clin Immunol.

[170] Martin A, Lavoie L, Goetghebeur M, Schellenberg R (2013). Economic benefits of subcutaneous rapid push versus intravenous immunoglobulin infusion therapy in adult patients with primary immune deficiency. Transfus Med.

[171] Gerth WC, Betschel SD, Zbrozek AS (2014). Implications to payers of switch from hospital-based intravenous immunoglobulin to home-based subcutaneous immunoglobulin therapy in patients with primary and secondary immunodeficiencies in Canada. Allergy Asthma Clin Immunol.

[172] Carmo EV, Correa M, Mazzucchelli JL, Tavares L, Damasceno E, Costa-Carvalho BT (2015). Socioeconomic impact of immunoglobulin replacement therapy for primary immunodeficiency patients on the health public system in Brazil: a single center study. Value Health.

[173] Shapiro R (2013). Why I use subcutaneous immunoglobulin (SCIG). J Clin Immunol.

[174] Ochs HD, Lee ML, Fischer SH, Delson ES, Chang BS, Wedgwood RJ (1987). Self-infusion of intravenous immunoglobulin by immunodeficient patients at home. J Infect Dis.

