# *Erratum:* II Brazilian Consensus on the use of human immunoglobulin in patients with primary immunodeficiencies

**DOI:** 10.1590/S1679-45082017AE3844E

**Published:** 2017

Ekaterini Simões Goudouris ^1^ ,

Almerinda Maria do Rego Silva ^2^ ,

Aluce Loureiro Ouricuri ^3^ ,

Anete Sevciovic Grumach ^4^ ,

Antonio Condino-Neto ^5^ ,

Beatriz Tavares Costa-Carvalho ^6^ ,

Carolina Cardoso de Mello Prando ^7^ ,

Cristina Maria Kokron ^8^ ,

Dewton de Moraes Vasconcelos ^8^ ,

Fabíola Scancetti Tavares ^9^ ,

Gesmar Rodrigues Silva Segundo ^10^ ,

Irma Cecília Douglas Paes Barreto ^11^ ,

Mayra de Barros Dorna ^8^ ,

Myrthes Anna Maragna Toledo Barros ^8^ ,

Wilma Carvalho Neves Forte ^12^ ; in name of Primary Immunodeficiency Group of the Brazilian Society of Allergy and Immunology

^1^ Universidade Federal do Rio de Janeiro, Rio de Janeiro, RJ, Brazil.

^2^ Universidade Federal de Pernambuco, Recife, PE, Brazil.

^3^ Hospital Servidores do Estado do Rio de Janeiro, Rio de Janeiro, RJ, Brazil.

^4^ Faculdade de Medicina do ABC, Santo André, SP, Brazil.

^5^ Universidade de São Paulo, São Paulo, SP, Brazil.

^6^ Universidade Federal de São Paulo, São Paulo, SP, Brazil.

^7^ Hospital Infantil Pequeno Príncipe, Curitiba, PR, Brazil.

^8^ Hospital das Clínicas, Faculdade de Medicina, Universidade de São Paulo, São Paulo, SP, Brazil.

^9^ Hospital de Base do Distrito Federal, Brasília, DF, Brazil.

^10^ Universidade Federal de Uberlândia, Uberlândia, MG, Brazil.

^11^ Centro Universitário do Estado do Pará, Belém, PA, Brazil.

^12^ Faculdade de Ciências Médicas, Santa Casa de São Paulo, São Paulo, SP, Brazil.

In the manuscript “II Brazilian Consensus on the use of human immunoglobulin in patients with primary immunodeficiencies”, DOI number: 10.1590/S1679-45082017AE3844, published at einstein (São Paulo). 2017; 15(1):1-16.

Page 6, **stated**: In this setting, it is possible to inject subcutaneous Ig every 21 to 28 days, just like intramuscular injections, with appropriate safety and good clinical results. ^(19)^ **It should be read:** In this setting, it is possible to inject subcutaneous Ig every 21 to 28 days, just like intravenous injections, with appropriate safety and good clinical results. ^(19)^

It the same article, page 8, **stated**: In chart 8, at the Osmolarity column, 32±4.5mOsmI/kg. **It should be read:** In chart 8, at the Osmolarity column, 327±4.5mOsmI/kg.

